# Ultrasound‐Guided Nerve Hydrodissection for Sural Nerve Injury: A Case Report and Review of the Literature

**DOI:** 10.1002/ccr3.72756

**Published:** 2026-06-08

**Authors:** Mengshan Zhu, Qiang Duan, Liangwen Sun, Fengli Yu, Chunxia Wei, Yumin Wang, Junjie Jian, Bo Wang, Xiaoqun Huang

**Affiliations:** ^1^ Department of Rehabilitation Medicine The First College of Clinical Medical Science, China Three Gorges University; Yichang Central People's Hospital Yichang China

**Keywords:** electromyography, hydrodissection, sural nerve, ultrasound

## Abstract

Sural nerve entrapment is a relatively uncommon cause of lateral ankle and foot pain and sensory disturbance. Conventional management generally lacks direct, targeted intervention at the site of nerve injury. We report a case of a 33‐year‐old male patient who developed sural neuropathy secondary to a right ankle sprain. Diagnosis was confirmed by electromyography (EMG) and high‐resolution musculoskeletal ultrasonography (US). The patient underwent two sessions of ultrasound‐guided perineural hydrodissection, achieving over 80% pain relief posttreatment, with sustained stability during follow‐up. This case highlights the diagnostic synergy of EMG and US in peripheral nerve entrapment syndromes, enabling precise diagnosis and anatomical localization of the entrapment site. It also supports ultrasound‐guided hydrodissection as a precise, safe, minimally invasive, and effective therapeutic intervention that may obviate the need for invasive surgery.

## Key Clinical Message

The combined application of electromyography and high‐resolution ultrasound enables accurate diagnosis of sural nerve entrapment, demonstrating significant diagnostic synergy. Ultrasound‐guided nerve hydrodissection is a precise, safe, and effective treatment particularly in patients refractory to conventional conservative measures.

**Abbreviations**
D5W: 5% dextrose in water
EMG: electromyography
MRI: magnetic resonance imaging
NPRS: Numeric Pain Rating Scale
SN: sural nerve
US: ultrasonography
VAS: Visual Analogue Scale

## 1 Introduction

The sural nerve (SN) originates from the convergence of the medial sural cutaneous nerve (a branch of the tibial nerve) and the lateral sural cutaneous nerve (a branch of the common peroneal nerve) in the distal to the mid‐calf region, providing sensory innervation to the posterior calf, lateral malleolus, and lateral border of the foot. As a form of peripheral nerve damage, the diagnosis and management of sural nerve injury have long faced challenges related to imprecise localization and the risks associated with invasive interventions. Advancements in high‐resolution musculoskeletal ultrasound technology have enhanced real‐time visualization of peripheral nerves, facilitating both accurate diagnosis and image‐guided interventions. Ultrasound‐guided perineural hydrodissection—defined as the targeted injection of fluid to mechanically separate an entrapped nerve from surrounding adhesive tissue—has emerged as a promising, evidence‐informed approach for managing compressive neuropathies. This paper presents a well‐documented case of sural nerve entrapment successfully managed with ultrasound‐guided nerve hydrodissection, supplemented with a literature review, to serve as reference for the precise diagnosis and management of such conditions.

## 2 Case Presentation

### 2.1 History of Present Illness

A 33‐year‐old male patient was admitted to the hospital with a 3‐week history of progressive numbness and pain localized to the lateral aspect of the right foot and ankle. Symptoms commenced acutely following an ankle sprain sustained on October 20, 2025. Although active ankle dorsiflexion was preserved, weight‐bearing ambulation was significantly impaired due to sharp, movement‐evoked pain upon heel strike. Initial outpatient management—including nonsteroidal anti‐inflammatory drugs and analgesic medications—yielded minimal symptomatic relief; the patient presented to our hospital on November 11, 2025. Electromyography indicated “right lower extremity sural nerve impairment,” and he was subsequently hospitalized with a diagnosis of sural nerve injury.

### 2.2 Past Medical History

The patient had no significant past medical history other than recurrent ankle sprains and denied comorbidities such as hypertension or diabetes.

### 2.3 Specialized Physical Examination

**FIGURE 1:**
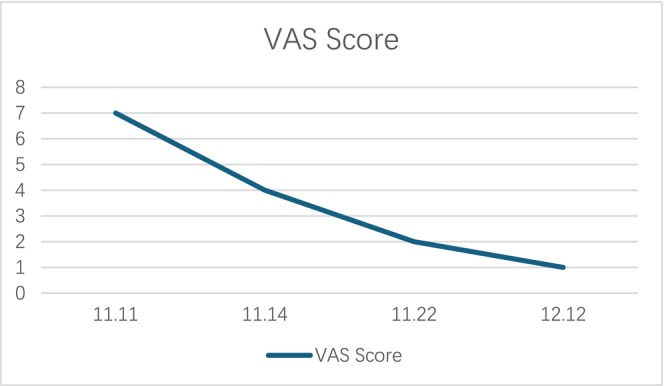
VAS Score.

No swelling, ecchymosis, or skin changes were observed over the right ankle or foot. Tenderness was positive (+) over the posterolateral right ankle, with a Visual Analogue Scale (VAS) score of 7 (Figure 1). Numbness was noted on the lateral aspect of the right foot dorsum. Muscle strength and range of motion of the right ankle and toes were Grade 5/5 and within normal limits, respectively. Dorsalis pedis artery pulsation was palpable. Examinations of the lumbar spine (straight leg raise test and reinforcing test, etc.) were negative. Bilateral patellar and Achilles tendon reflexes were symmetrical, and no pathological reflexes were elicited.

### 2.4 Auxiliary Examination

**FIGURE 2:**
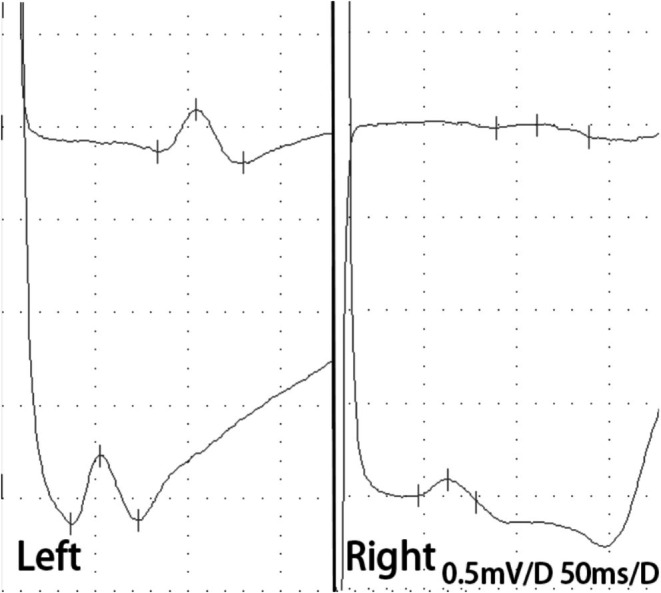
Electromyography results. The conduction of the left SN is normal; the conduction of the right SN is slowed, with a decrease in amplitude.

Electromyography (November 11, 2025): Demonstrated reduced amplitude and delayed latency of the right sural sensory nerve action potential (Table 1; Figure 2).

**TABLE 1:** Electromyography results of the SN.

Nerve	Stimulation site	Lat (ms)	Amp (μV)	Dist (mm)	CV (m/s)	Stim (mA)
Left SN	From mid‐calf to lateral malleolus	3.33	11.4	170	51.1	27.4
	From lower mid‐calf to lateral malleolus	1.46	13.9	70	47.9	13.2
Right SN	From mid‐calf to lateral malleolus	3.56	2.6	170	47.8	30.0
	From lower mid‐calf to lateral malleolus	1.89	4.8	70	37.0	14.4

**FIGURE 3:**
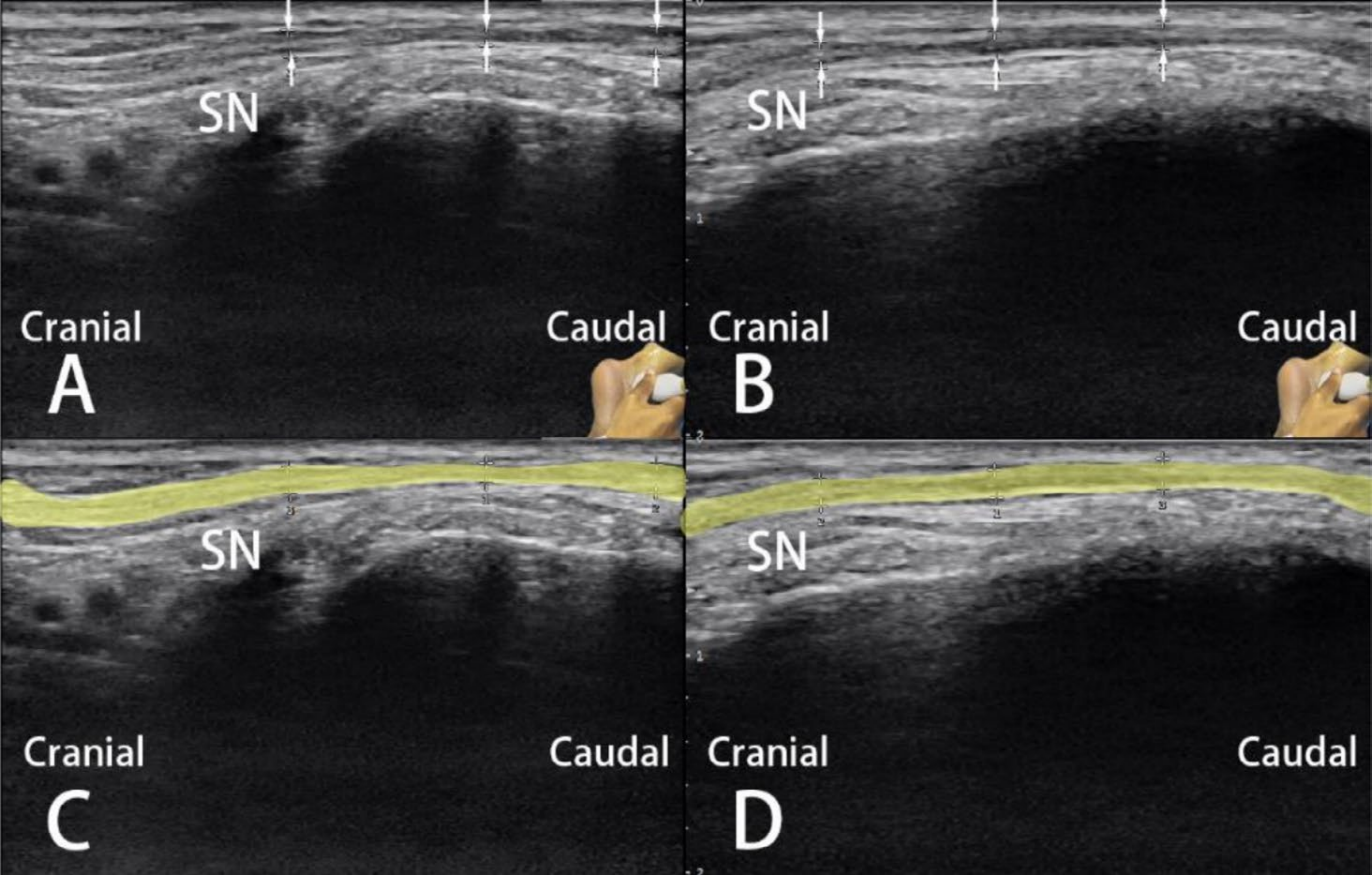
Musculoskeletal ultrasound examination images. (A, C) Long‐axis images of the sural nerve (SN) before the first treatment (November 12, 2025); (B, D) Long‐axis images of the nerve after the first treatment (November 22, 2025).

Musculoskeletal Ultrasound (November 12, 2025): Revealed focal hypoechogenicity and caliber reduction (diameter: 1.1 mm) of the right sural nerve at the level of the lateral malleolus, contrasting with proximal (1.5 mm) and distal (1.6 mm) segments (Table 2; Figure 3). Overall Impression: Suspicion of entrapment neuropathy at the lateral malleolus segment of the right sural nerve.

**TABLE 2:** Ultrasound Assessment of the Long‐Axis Diameter of the SN at the Level of the Ankle.

Time	Cranial (mm)	Narrow (mm)	Caudal (mm)
Before the first treatment (November 12, 2025)	1.5	1.1	1.6
After the first treatment (November 22, 2025)	1.4	1.3	1.5
After the second treatment (December 12, 2025)	1.4	1.4	1.6

Contrast‐Enhanced MRI of the Right Calf (November 13, 2025): No structural abnormalities, mass lesions, or inflammatory signal changes were identified within the nerve or adjacent soft tissues.

Based on the patient's medical history, symptoms, and auxiliary examinations, the diagnosis was sural nerve injury.

### 2.5 Treatment

During hospitalization, initial conservative management—including topical diclofenac diethylamine emulgel, oral mecobalamin, vitamin B1, pregabalin, and comprehensive physical rehabilitation (low‐frequency pulsed electrotherapy, ultrasonic therapy, microwave therapy, traditional Chinese medicine fomentation therapy, etc.)—failed to produce clinically meaningful improvement. Following formal consent and exclusion of contraindications (e.g., infection, coagulopathy, allergy to injectate), ultrasound‐guided perineural hydrodissection was performed on November 14, 2025.

### 2.6 Operative Procedure and Results

**FIGURE 4:**
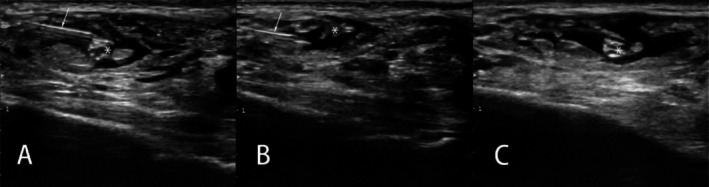
Process diagrams of sural nerve hydrodissection. (A) Superficial dissection of the sural nerve (*), with the arrow indicating the needle tip; (B) Deep dissection; (C) Short‐axis view of the nerve after hydrodissection.

Under ultrasound guidance, the lateral malleolar segment of the sural nerve was located, and an in‐plane needle approach was used. A mixture of 2 mL each was injected into the deep and superficial fascial spaces around the nerve in the short‐axis view, containing 5% dextrose in water (D5W), mecobalamin, lidocaine, and betamethasone. Postinjection ultrasound demonstrated fluid enveloping the nerve, with separation of the nerve from the surrounding fascial tissues observed in the long‐axis view, confirming successful hydrodissection (Figure 4). Postprocedure, the patient reported moderate improvement in pain and numbness over the lateral malleolus (VAS score: 4) and requested discharge.

### 2.7 Follow‐Up

**FIGURE 5:**
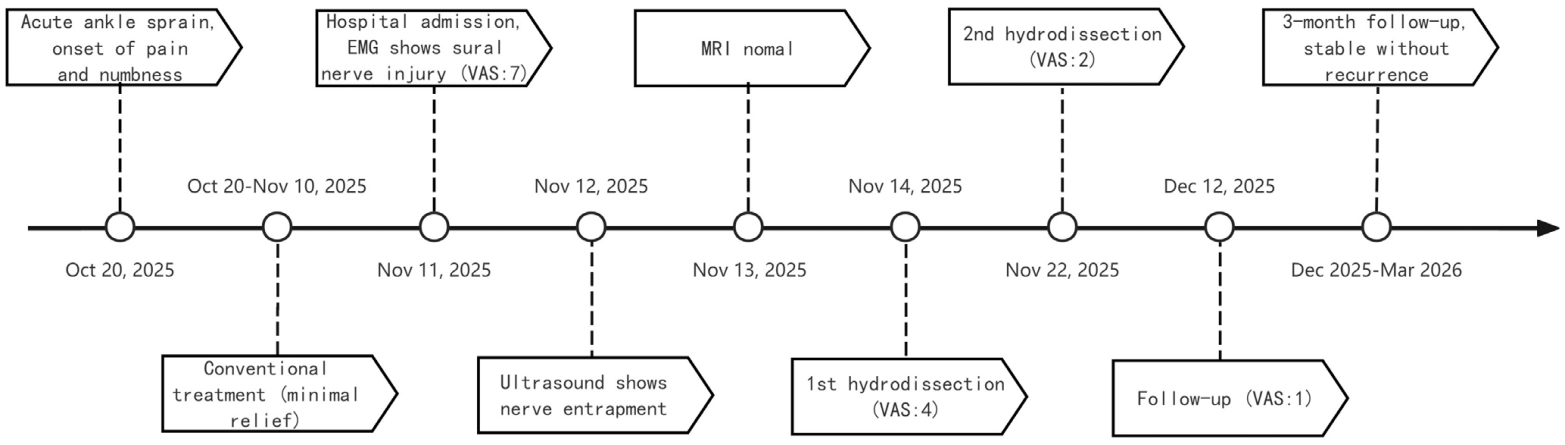
Timeline chart of the entire diagnosis and treatment process.

The patient subsequently attended multiple outpatient follow‐ups and underwent a second ultrasound‐guided hydrodissection on November 22, 2025, after which the VAS score decreased to 2. At the follow‐up on December 12, 2025, significant pain relief with only occasional dorsolateral foot numbness was reported (VAS score: 1). Symptoms remained consistently stable without recurrence during the 3‐month follow‐up period. A timeline chart of the entire diagnosis and treatment process is presented (Figure 5).

## 3 Discussion

The sural nerve is a purely sensory peripheral nerve that courses inferoposteriorly alongside the small saphenous vein and the lateral border of the Achilles tendon, providing sensory innervation to the distal third of the posterior calf, the lateral malleolus, the dorsolateral foot, and the fifth toe. Entrapment or focal injury of this nerve most commonly arises from mechanical compression—such as repetitive ankle trauma, postsurgical scarring, fascial tethering, or iatrogenic injury—manifesting clinically as neuropathic pain, dysesthesia, and sensory loss within its dermatomal distribution [1]. In the present case, the patient's history of recurrent ankle sprains, coupled with symptom localization and physical examination findings, strongly suggested focal sural neuropathy.

Electromyography (EMG) remains the electrophysiological gold standard for confirming sural nerve involvement and differentiating it from proximal neuropathies (e.g., common peroneal or tibial nerve compression), thereby informing both diagnostic certainty and prognostic stratification [2]. Magnetic resonance imaging (MRI) lacks sufficient spatial resolution to reliably detect subtle morphological changes, such as focal nerve narrowing, perineural fibrosis, or early edema; however, it offers excellent soft‐tissue contrast and is indispensable for excluding structural mimics, including fractures, soft‐tissue masses, neuromas, or inflammatory lesions [3]. High‐resolution musculoskeletal ultrasonography provides dynamic, real‐time assessment of peripheral nerves, enabling precise evaluation of cross‐sectional area, echotexture, fascicular architecture, and spatial relationships with adjacent anatomical structures. Characteristic sonographic features include a hypoechoic “honeycomb” pattern in short‐axis view and a longitudinally striated “corduroy” appearance in long‐axis view [4]. Pathological alterations—such as focal hypoechogenicity, caliber reduction with proximal/distal swelling, and loss of normal fascicular definition—are highly suggestive of entrapment [5]. In this case, EMG confirmed sural nerve injury; while MRI revealed no significant abnormalities and excluded fractures, neuromas, and other pathologies; and US demonstrated focal caliber reduction (1.1 mm) at the lateral malleolar segment, accompanied by relative swelling proximally and distally—collectively supporting a diagnosis of localized sural nerve entrapment.

Conventional conservative management relies on systemic anti‐inflammatory and analgesic medications, neuropathic pain medications, neurotrophic agents, and multimodal physical rehabilitation, but does not target the underlying mechanical compression. Ultrasound‐guided perineural hydrodissection represents a pathophysiologically grounded intervention: by injecting a small volume of fluid into the epineural or interfascial space, it mechanically separates the nerve from restrictive adhesions or compressive fascial bands, mitigates perineural inflammation, and enhances intraneural microcirculation [6]. Crucially, real‐time ultrasound guidance ensures accurate needle placement, enables visualization of injectate dispersion around the nerve (“separation sign”), and minimizes risks of intraneural injection or vascular puncture—thereby optimizing procedural safety, reproducibility, and therapeutic precision [7]. This technique has demonstrated efficacy across multiple peripheral nerve entrapment syndromes, including carpal tunnel syndrome [8] and sciatic nerve entrapment [9].

The technical steps for ultrasound‐guided nerve hydrodissection typically encompass preprocedural preparation, ultrasound localization, needle puncture and injection, and postprocedural functional reassessment. In this case, a high‐frequency linear ultrasound probe was first used to trace the entire course of the sural nerve from the lateral head of the gastrocnemius to the area posterior to the lateral malleolus. Short‐axis imaging revealed focal hypoechogenicity and reduced caliber at the lateral malleolus, while long‐axis views confirmed focal stenosis with preserved continuity—consistent with mechanical entrapment rather than transection or neuroma formation. During injection, an in‐plane needle approach may be employed. Following hydrodissection, real‐time US documented circumferential fluid accumulation and separation of the nerve from surrounding connective tissue planes. Postprocedurally, neurological function was assessed immediately, and a follow‐up ultrasound examination was performed 1–2 weeks later to reevaluate structural resolution [10]. Rapid and sustained symptomatic relief was reported—supporting the biological plausibility of mechanical decompression as the primary mechanism of action.

In recent years, although ultrasound‐guided peripheral nerve interventions have gained widespread acceptance in interventional neurology and sports medicine, evidence specific to sural nerve hydrodissection remains sparse. A systematic literature review conducted across PubMed, MEDLINE, Cochrane Library, Web of Science, and ScienceDirect—using the terms “Sural Nerve,” “ultrasound,” and “hydrodissection,” —identified six published case reports meeting inclusion criteria (Table 3).

**TABLE 3:** Comparison of cases of sural nerve injury treated with ultrasound‐guided neural hydrodissection.

Case	Cause	Treatment	Follow‐up
Omodani T and Takahashi K Case [11]	Combination of scar tissue and the physical presence of a fixation plate after calcaneus fracture surgery	Hydrodissection (5 mL of 0.09% lidocaine diluted in saline) at the lateral side, distal end of the calcaneus and subsequent surgery (plate removal and neurolysis)	Pain relieved (10 to 3 on numerical rating scale) for 10 months after hydrodissection. Pain disappeared completely after plate removal and neurolysis.
Yoon Y and Lam KHS Case [12]	Scar tissue persisting after calcaneal fracture repair and subsequent hardware removal	Two sessions of hydrodissection (20 mL 5% dextrose in water (D5W))	Sustained over 90% symptom resolution was reported (VAS score improved from 9/10 to 1/10) after the second session at 6‐month follow‐up.
Yoon Y and Lam KHS Case [13]	Fascial tissues affected by Psoriasis	Hydrodissection (50 mL of 5% dextrose in water (D5W)) at the lateral calf region	Immediate functional improvement (NPRS reduction from 8 to 2 during ambulation) and complete resolution of Achilles stiffness were reported, with symptoms remaining stable throughout 24‐month follow‐up.
Lotliker SD and Verma S Case [14]	Haglund's deformity	Left ankle injection (a mixture of 0.5% bupivacaine 20 mg + dexamethasone 4 mg + 250 mg amikacin, total volume 5 mL) at the site of pain, near the superficial branches of the sural nerve	At 1‐year postprocedure follow‐up, the patient still had sustained 100% pain relief (VAS score improved from 7/10 to 0/10).
Fader RR and Mitchell JJ Case [15]	Sural nerve entrapment with edema and neuromatous scar formation due to a history of recurrent training‐induced right‐sided gastrocnemius strains	Hydrodissection (with 2 mL 1% lidocaine, 1 mL betamethasone acetate/betamethasone sodium phosphate, and 7 mL 5% dextrose) at the epineurium of the sural nerve at the point of entrapment	The patient had complete relief of symptoms and full return to preinjury level of participation in competitive sports.
Liu I‐C and Wu C‐H Case [16]	A hyperpigmented scar secondary to a wound from a traffic accident	Two sessions of hydrodissection (5% dextrose (5 mL))	“Tense sensation” was improved and the skin pigmentation faded as well. Additionally, the SN cross‐sectional area was reduced from 0.07 to 0.04 cm^2^.

## 4 Conclusion

Although the etiologies of sural nerve entrapment vary, all reported favorable outcomes following neural hydrodissection, with symptoms maintained or markedly improved during follow‐up. This technique offers an effective interventional option for patients who have failed conservative treatment and wish to avoid surgery. However, further validation through larger scale clinical studies is warranted.

## Author Contributions

**Mengshan Zhu:** writing – original draft. **Qiang Duan:** supervision, funding acquisition, investigation. **Liangwen Sun:** project administration, supervision. **Fengli Yu:** investigation. **Chunxia Wei:** writing – review and editing. **Yumin Wang:** writing – review and editing. **Junjie Jian:** writing – original draft. **Bo Wang:** writing – review and editing. **Xiaoqun Huang:** conceptualization.

## Funding

This work was supported by the Natural Science Foundation of Hubei Province (Joint Fund Program) (No.2024AFD137).

## Disclosure

Declaration of large language models: No large language models were used in manuscript writing.

## Ethics Statement

As a single‐case report with the patient's signed consent, no additional ethical review was required.

## Consent

Written informed consent was obtained from the patient for publication of this case report.

## Conflicts of Interest

The authors declare no conflicts of interest.

## Data Availability

The data that support the findings of this study are available from the corresponding author upon reasonable request.
